# Further validation of the patient roles and responsibilities scale

**DOI:** 10.1080/21642850.2026.2711472

**Published:** 2026-07-31

**Authors:** Valerie Shilling, Rebecca Foster, Lynn Calman, Claire Foster

**Affiliations:** a Sussex Health Outcomes, Research and Education in Cancer, Brighton and Sussex Medical School, University of Brighton and University of Sussex, Brighton, UK; b Centre for Psychosocial Research in Cancer: CentRIC+, School of Health Sciences, University of Southampton, Southampton, UK

**Keywords:** Cancer, patient reported outcome measures, psychometric performance, quality of life, confirmatory factor analysis

## Abstract

**Background:**

The Patient Roles and Responsibilities Scale (PRRS) measures the impact of cancer and treatment on ‘real world’ functioning such as caregiving and financial responsibilities. It was developed and validated in advanced cancer but with the intention to be sufficiently generic and appropriate for use in all cancers and stages. Here we further investigate the psychometric performance of the PPRS when completed by people three years after cancer diagnosis, treated with curative intent.

**Methods:**

This is a secondary analysis of data collected during the HORIZONS Programme, a prospective longitudinal cohort study of the impact of cancer and treatment on everyday life. Data analysed were from the 36-month timepoint and included the PRRS alongside the Work and Social Adjustment Scale, the Quality of Life in Adult Cancer Survivors and the EORTC QLQ-C30. Internal consistency was assessed using Cronbach's alpha, criterion and convergent validity through correlation with other quality-of-life measures, and construct validity using confirmatory factor analysis (CFA).

**Results:**

Data from 1077 participants (453 breast, 388 gynaecological, 236 non-Hodgkin lymphoma) were included in the analysis. Cronbach's alpha was 0.91 for the PRRS-16 and 0.69–0.92 for the subscales. The measure showed the predicted pattern of correlation with validation measures r = |0.58–0.82|. All CFA goodness-of-fit criteria were met, indicating an acceptable fit for the three-factor model, though correlations between factors were stronger than expected.

**Conclusion:**

This study provides further evidence of the reliability and validity of the PRRS and its performance and appropriateness for use in cancer groups other than those previously validated.

## Background

In recent decades, rapid advances in cancer treatment have resulted in many more thousands of people living with and beyond cancer. This, in turn, has widened the focus of measurement of cancer quality of life to include a broader assessment of wellbeing, alongside symptom and side effect monitoring (Fallowfield et al., 2017).

The Patient Roles and Responsibilities Scale (PRRS) was developed specifically to enable a broader evaluation of the impact of cancer and its treatment, measuring ‘real world’ role functioning such as caring for others, and worries about finances and employment (Shilling et al., 2018). It is intended to be of use for both research and practice, such as to aid clinical conversations around treatment and intervention.

The development and validation of the PRRS has been previously described (Shilling et al., 2018). The scale showed good internal consistency, test‒retest reliability, criterion and convergent validity, and sensitivity to change. Principal component analysis suggested a three-factor solution accounting for 61.5% of total variance. These three factors were proposed as useful subscales, measuring responsibilities and social life, family wellbeing, and financial wellbeing, respectively, and continue to form the structure of the measure today.

The PRRS sits within the FACIT measurement system (www.facit.org). Alongside the English version, official FACIT translations are available in German and are underway in Portuguese and Chinese. It has been used in a number of research projects e.g. Fallowfield et al. (2023); Harder et al. (2024); Jenkins et al. (2023), including HORIZONS (Foster et al., 2019).

The Macmillan HORIZONS Programme (Foster et al., 2019) was a prospective longitudinal cohort study which aimed to examine the impact of cancer and its treatment on everyday life, and the quality of that life. Participants completed baseline questionnaires prior to the start of treatment, which was being given with curative intent, even if the chances of cure were low. They were followed up at set intervals over a number of years, to better understand the impact of cancer treatment on recovery over time. The study comprised three cohorts: breast, gynaecological and non-Hodgkin lymphoma. The breast cancer cohort included 1394 women under 50 at the time of diagnosis. The gynaecological cohort included 1223 women with cervical, endometrial, ovarian or vulvar cancer. The non-Hodgkin lymphoma cohort included 690 men and women. A wide range of clinical, sociodemographic and patient-reported psychosocial and quality of life measures were included. The PRRS was included at the 36-month time point.

The PRRS was developed and initially validated with patients with advanced cancer (breast, lung, gynaecological cancer or melanoma); however, the measure was developed with the intention to be sufficiently generic to apply to all disease stages and cancer types. The scale of the HORIZONS Programme provides an important opportunity to further evidence the reliability and validity of the measure in a much larger group of patients three years post-diagnosis who were treated with curative intent (even if the chances of cure were low), and include a novel cancer group, non-Hodgkin lymphoma. The objectives of the present study are to further demonstrate the reliability (internal consistency) and validity (criterion, convergent and construct) of the PRRS.

## Methods

### Participants and procedure

Data for this secondary analysis were taken from the 36-month time point of the HORIZONS Programme. All cohorts were included. Only data from participants with a valid PRRS total score were included in the analyses (more than 80% of items complete overall and valid scores for each subscale, requiring more than 50% of items complete per subscale).

Written informed consent for the HORIZONS Programme was provided by all participants, including consent for anonymised data to be used in future research. The HORIZONS Programme received ethics and Health Research Authority approval (REC reference number 16/NW/0425). This secondary analysis of the HORIZONS data received ethics approval from the Brighton and Sussex Medical School Research Governance and Ethics Committee (ref ER/BSMS6375/3). Data were shared between the Universities of Southampton and Sussex under a data sharing agreement. The dataset contained no information that could identify individual participants.

### Measures

#### Patient Roles and Responsibilities Scale (PRRS)

The PRRS (Shilling et al., 2018) was developed to measure the impact of treatment on patients' roles and responsibilities such as caregiving, family, financial and occupational responsibilities. It comprises three core subscales: responsibilities and social life (five items), family wellbeing (five items), financial wellbeing (six items), and a standalone subscale jobs and career (seven items) completed only by patients in current employment (including those on long-term sick leave). The PRRS sits within the functional assessment of chronic illness therapy (FACIT) measurement system. It requires Likert-type responses ranging from ‘not at all’ to ‘very much’ enquiring how much of a problem or how bothered patients have been by specific items in the past 7 days. A higher score indicates better quality of life. The scale in full is available as a Supporting Information file; please note permission for use must be sought prior to use.

#### Work and Social Adjustment Scale (WSAS)

The Work and Social Adjustment Scale (Marks, 1986) has five items assessing impairment on work, home management, social leisure activities, private leisure activities and family and relationships. Participants rate how much each activity is impaired as a result of an identified problem, in this case, cancer. Items are scored on a scale of 0 (no impairment) to 8 (very severe impairment). The questionnaire is scored by totalling responses to the five items, with a total score of 40, with higher scores indicating greater impairment.

#### Quality of Life in Adult Cancer Survivors (QLACS)

The QLACS (Avis et al., 2006) contains 47 items grouped into 12 domains grouped as generic (negative feelings, positive feelings, cognitive problems, sexual problems, physical pain, fatigue and social avoidance) and cancer specific (financial problems, family-related distress, distress about recurrence, appearance concerns, and benefits from cancer). Items are scored on a scale of 1 (*never*)-to-7 (*always*), with lower scores (i.e. more frequent reports of ‘never’) corresponding to higher quality of life. Summary score can be calculated for both the generic and cancer-specific domains. Higher scores indicate better quality of life.

#### EORTC QLQ-C30

The EORTC *QLQ-C30* (Aaronson et al., 1993) has 30 items measuring functional status (physical, social, emotional, role and cognitive), symptoms (such as nausea and vomiting, fatigue, breathlessness) and single item measures of global health and quality of life. Two summary scores can be calculated using (1) the functional and symptom questions (excluding financial difficulties) and (2) the global health and quality of life items.

#### Sociodemographic variables

Sociodemographic data included: age at consent, ethnicity, sex, relationship status, caring responsibilities, employment status and highest level of education.

### Analysis

Analyses were conducted using the Statistical Package for Social Sciences (IBM SPSS; version 29.0.2) and R version 4.4.1 (R Core Team, 2021) using the Lavaan package (Rosseel, 2012).

Missing data were managed as per the specific instrument guidance. For the PRRS, questionnaire completion is only valid if 80% of items overall are completed. Each subscale score may be prorated if more than 50% of items are answered. A total score may only be calculated if each subscale has a valid score.

PRRS data quality was assessed by consideration of the missing data overall and per item. 15% was set as the acceptable threshold for individual items. Floor/ceiling effects per item were considered (threshold > 70%), as was an absolute skew of >|2|. Absolute value was used rather than z-score due to the large sample size (West et al., 1995).

Internal consistency of the PRRS total and subscales was assessed using Cronbach's alpha (for good internal consistency, α ≥ 0.7 is expected). Corrected item total correlations (CITC) were reviewed for each subscale with each item expected to have CITC > 0.3.

Criterion validity was assessed by correlation of the PRRS total score with a commonly used measure of similar concepts, the Work and Social Adjustment Scale. A strong negative correlation (*r* > −0.7) was predicted. Convergent validity was further evaluated by correlation with other similar measures. We predicted a moderate to strong negative correlation (−0.5 < *r* < −0.7) between PRRS total and QLACS, and a moderate to strong positive correlation (0.5 < *r* < 0.7) between PRRS total and QLQC30.

The confirmatory factor analysis model was specified using the 16 core scale items of the PRRS with three latent variables specified, corresponding to the three subscales: responsibilities and social life (five items), family wellbeing (five items), and financial wellbeing (six items).

The model was estimated using weighted least squares mean and variance adjusted (WLSMV) estimator given the ordinal nature of the data. Latent factors were constrained and missing data were managed by listwise deletion. Goodness of fit was evaluated based on the common fit indices (and thresholds for adequate fit): root mean square error of approximation (RMSEA ≤ 0.08), standardised root mean square residual (SRMR ≤ 0.08), comparative fit index (CFI ≥ 0.9), and Tucker-Lewis index (TLI ≥ 0.9). We chose a priori not to include the χ^2^ statistic as a key indicator, given the dependence of this test on sample size; specifically, because it may reject appropriate models in larger sample sizes such as this as it readily reaches significance.

## Results

### Participants

Of the 3307 participants recruited to the HORIZONS Programme, 1077 had valid PRRS data at 36 months and are included in this analysis. A total of 453 (42%) had a diagnosis of breast cancer, 388 (36%) gynaecological cancer and 236 (22%) non-Hodgkin lymphoma. Mean age was 58 years (S.D. 13.37), with a range of 25 to 90. Given the nature of the included cancers, the majority of participants were female (932 [88%] vs 131 [12%]). Further participant characteristics are shown in Table 1.

**Table 1.** Participant characteristics.

***Age in years at consent, N = 1077***	***Mean (SD)* 58 (13.37) *Median, range* 56, 25–90 years**
*Cancer type, N = 1077*	*n (%)*
Breast	453 (42)
Gynaecological^a^	388 (36)
Non-Hodgkins Lymphoma	236 (22)
*Ethnicity, N = 1052*	*n (%)*
White British	970 (92)
White other	48 (5)
Non-White^b^	34 (3)
*Sex, N = 1063*	*n (%)*
Female	932 (88)
Male	131 (12)
*Relationship status N = 1017*	*n (%)*
With partner	791 (78)
No partner^c^	226 (22)
*Caring responsibilities for under 18 s, N = 862*	*n (%)*
Yes	248 (29)
No	614 (71)
*Employment status N = 1077*	*n (%)* ^d^
Full-time	247 (23)
part-time work	214 (20)
Self-employed	86 (8)
Unemployed	28 (3)
Disabled or long-term sick leave	32 (3)
On maternity leave	2 (<1)
Retired	412 (38)
In full or part-time education or training	7 (<1)
Looking after home or family	48 (4)
Voluntary work	17 (2)
Other	4 (<1)
*Highest level of education N = 892*	*n (%)*
Compulsory school education or less	130 (15)
Apprenticeship or further education	168 (19)
Higher education undergraduate	150 (17)
Higher education postgraduate	119 (13)
Professional qualifications	159 (18)
Other vocational/work-related qualifications	111 (12)
None of the above or other	55 (6)

^a^ Includes: endometrial, ovarian, cervical, and vulval

^b^ Includes: African, Asian, Black-Caribbean, Mixed, and Other

^c^ Includes: Single, divorced/separated, and widowed

^d^ Totals to more than 1077/100% as participants could tick multiple options

### PRRS data quality and descriptive statistics

#### Data quality: acceptability and precision

Percentage of missing data was low; the highest per item was 1.9% and across the PRRS data was 0.1%. Six of the sixteen items exceeded the 70% ceiling threshold and had an absolute skew > |2|. No items demonstrated floor effects.

Total scores on the PRRS Core-16 (*N* = 1077) ranged from 4 to 64 (possible range 0–64) with mean 53.0, SD 11.4, and skewness −1.4(SE = 0.75). One-hundred and thirty-one participants (12.2%) reported a maximum score (threshold for ceiling effects set at 15%); no participants had a minimum score.

### Internal consistency

Cronbach's *α* for the PRRS total score (*N* = 1013, listwise deletion based on all variables) was 0.91 (CITC range 0.38–0.77). Reliability analysis was also conducted for the three subscales. Cronbach's *α* for responsibilities and social life was 0.92 (*N* = 1063, CITC range 0.72–0.85); for family wellbeing *α* = 0.91 (*N* = 1048, CITC range 0.72–0.86), and for financial well-being *α* = 0.69 (*N* = 1056, CITC range 0.35–0.56). Inter-item and inter-subscale correlations are shown in Table 2.

**Table 2.** Inter-item correlations and correlations between subscale scores.

Subscale	Mean (SD) range *N* = 1077	Inter-item correlation range	Correlations between subscale scores		
			Responsibilities and social life	Family well-being	Financial well-being
Responsibilities and social life (five items, possible score 0–20)	17.00 (4.61) 0–20	0.62–0.79	1.00		
Family well-being (five items, possible score 0–20)	14.71 (5.36) 0–20	0.59–0.77	0.70	1.00	
Financial well-being (six items, possible score 0–24)	21.29 (3.71) 0–24	0.23–0.45	0.42	0.45	1.00

### Criterion and convergent validity

As predicted, total scores on the PRRS correlated strongly and negatively with scores on the WSAS (*N* = 1051, *r* = −0.82). PRRS scores also showed the predicted correlations with the QLACSSpecific (*N* = 1036, *r* = −0.65) and Generic (*N* = 997, *r* = −0.64) scales, and the QLQC30 Summary (*N* = 1051, *r* = 0.74) and Global (*N* = 1060, *r* = 0.58) scores.

### Confirmatory factor analysis

Listwise deletion resulted in 1013 included cases. All goodness-of-fit tests indicated an acceptable fit for the model: RMSEA = 0.028 (90% CI: 0.025,0.032), SRMSR = 0.052, robust CFI = 0.992, robust TLI = 0.991.

Table 3 shows the standardised factor loadings for each item against the latent variables. A standardised factor loading of ≥0.6 is considered sufficient to confirm that the latent variable adequately represents the observed variable (Hair et al., 1998). Thirteen of 16 variables load ≥0.6 on their latent variable; loadings range from 0.37 to 0.89. Moderate to strong correlations were found between factors one and two (*r* = 0.76), one and three (*r* = 0.54), and two and three (*r* = 0.57).

**Table 3.** Standardised factor loadings.

Item	Loading
**Factor 1: responsibilities and social life**	
My illness interferes with performing my responsibilities at home (e.g. cooking, cleaning, gardening, DIY)	0.82
I am less able to fulfil my caregiving responsibilities (e.g. looking after children, grandchildren, another adult, pets)	0.83
I have less patience for my caregiving responsibilities (e.g. looking after children, grandchildren, another adult, pets)	0.77
I feel sad that my illness forces me to miss out on doing things with my children and/or other family members	0.88
I socialise less because of my illness	0.86
**Factor 2: family well-being**	
I worry about the impact of my illness on my partner (or the person who is my main support)	0.81
I worry about the impact of my illness on my children and/or other family members	0.89
I worry about the impact of my illness on people that I normally provide support to (e.g. friends, neighbours, parents and/or grandchildren)	0.82
The way I see myself within the family has changed because of my illness	0.82
I worry how my family will cope in the future	0.79
**Factor 3: financial well-being**	
I feel in control of my financial situation	0.37
I worry about the financial problems I will have in the future as a result of my illness or treatment	0.72
My family and/or friends have to help me financially	0.51
My family gives up things because of the financial impact of my illness	0.57
The additional costs of my illness are more than I thought they would be (e.g. travel and parking, heating, healthy eating, supplements, non-prescription medication, paying for help at home)	0.71
I have difficulty meeting the additional costs of my illness	0.65

All factor loadings are statistically significant (*p* < 0.001).

## Discussion

The PRRS was originally validated with patients with advanced cancer (breast, lung, gynaecological cancer or melanoma). In the current study, we sought to further validate the measure in a larger group of patients from the HORIZONS Programme. Importantly, though a majority of this cohort also comprised patients who had a diagnosis of breast or gynaecological cancer, they were three years post-diagnosis and had been treated with curative intent.

The measure demonstrated good internal consistency, both for the total score and the individual subscales. Scores on the PRRS also performed as expected against other measures, demonstrating strong criterion and convergent validity. Different measures were used in the HORIZONS Programme to those in the original validation study, so we were unable to make direct comparisons.

We conducted confirmatory factor analysis to test the construct validity of the measure, using a model based on the three factors identified in earlier principal components analysis, on which the three subscales are based. All goodness-of-fit tests indicated an acceptable fit for the model, with all factor loadings significant and a majority achieving high loadings.

We anticipated an association between factors 1 (responsibilities and social life) and 2 (family wellbeing), as both contain items pertaining to (different) aspects of family life, with factor one being concerned with practical responsibilities and factor two more associated with emotional impact. The correlations between factors were higher than expected, however, suggesting that the subscales may not be measuring entirely distinct constructs and may be interpreted as related facets rather than fully independent constructs.

Missing data for the PRRS was low (0.1% overall), confirming that participants found the measure acceptable. Ceiling effects per item were relatively common (6/16 items). By contrast, only one item showed ceiling effects in the original validation with patients with advanced disease, likely reflecting the different time since diagnosis and prognosis between the two groups.

The observed ceiling effects may suggest that some items may have limited discriminative ability among respondents who are doing well, which is an important consideration when applying it in long-term survivorship populations. It may be that the measure is more suited to identifying individuals with ongoing difficulties, but less optimal for monitoring patients who are ‘doing well’, some time after a cancer diagnosis. Ceiling effects were more common in items relating to financial well-being, which may warrant further investigation.

Overall, the cohort in the current study reported higher total scores on the PRRS, indicating better well-being/lower impact, than the original validation sample (mean 53.33 [S.D. 11.23] vs 39.2 [13.04], maximum possible score 64).

### Limitations of the study

In the HORIZONS Programme, the PRRS was used only at the 36-month time point. As such, we were unable to examine test-retest reliability or sensitivity to change however, the size of the dataset has enabled a thorough investigation of the internal consistency and validity of the measure. In the original validation study, we demonstrated preliminary sensitivity to change of the PRRS using the anchor variable: I am content with the quality of my life right now (Shilling et al., 2018). Future research would be valuable to establish Minimally Important Differences (MIDs) for the measure and subscales, to define what is meaningful change to patients, increasing usability in a clinical setting.

Due to the nature of the cancers included in the HORIZONS Programme, a majority (88%) were female. While we have no a priori reason to suggest the measure would perform differently in men and women, or across cancer types, we acknowledge that this sample is not representative of sex, ethnicity, or cancer type and should be interpreted accordingly. The ethnic diversity of the sample does not reflect that of the UK population. Future research should explicitly seek to recruit a more representative sample. This may be particularly relevant as concepts such as caregiving and family wellbeing are culturally influenced and as such, it is important to demonstrate the psychometric properties of the measure in different groups.

A total of 3307 people were recruited to the HORIZONS programme. Only 1077 had valid data at 36 months. While this remains a large sample and more than sufficient for the planned analyses, we must consider the possibility that those who remained in the study may differ systematically from those who dropped out. It is possible that those remaining in the study had better health and higher functioning than those who dropped out, which may in turn have influenced the ceiling effects found. Retention in longitudinal studies can be challenging as some patients prefer not to be reminded about their cancer as they move beyond the initial diagnosis and treatment phase.

Of the 1077 participants included in the analysis, 439 completed the jobs and career subscale of the PRRS. This is a standalone scale, completed only by those in paid employment. It does not contribute to the PRRS core-16 total score. The jobs and career subscale was revised in 2024, based on additional data from cognitive interviews. The revision resulted in minor wording changes to three of the seven items and more significant changes to two others. Data from the HORIZONS Programme predate these changes and as a result, we were not able to use the data to look at the reliability of this standalone scale.

## Conclusion

A number of well-validated measures of real-world functioning are available in addition to those included in this analysis. These include the Comprehensive Score for Financial Toxicity (COST), a measure of financial distress (de Souza et al., 2014, 2017), the Work Productivity and Activity Impairment (WPAI) focussed on employment (Reilly et al., 1993), and the Parenting Concerns Questionnaire (Muriel et al., 2012) to name a few.

Perhaps most similar to the PRRS is the social difficulties inventory (SDI) measure of social distress (2011; Wright et al., 2008), which was used to assess criterion validity in the original PRRS validation study and contains items relating to everyday living and finances and well as practical difficulties and barriers to participation.

The PRRS is a comprehensive measure which was developed to measure multiple dimensions including family and home responsibilities, financial responsibilities and work and employment roles. We expand further on the need for the measure in our earlier publications (Catt et al., 2017; Shilling et al., 2018). The scale is intended to move beyond ‘can you function?’ to ‘are you able to fulfil your usual roles in life?’

The PRRS was developed to capture broad ‘real life’ impacts of cancer and its treatment. It is intended to be useful for both research and practice, for example, to facilitate clinical conversations around aspects of life not assessed in routine side effect monitoring. This study provides further evidence of the reliability and validity of the measure and its performance and appropriateness for use in cancer groups other than those previously validated.

## Data Availability

This is a secondary data analysis of data from the HORIZONS programme. It is not possible for the authors to make the original data publicly available. The data may be available to researchers to conduct further analysis via a data access request to the Centre for Psychosocial Research in Cancer (CentRIC+) CentRIC@soton.ac.uk and an appropriate institutional data sharing agreement.
